# Characterization of Thermo- and Detergent Stable Antigenic Glycosylated Cysteine Protease of *Euphorbia nivulia* Buch.-Ham. and Evaluation of Its Ecofriendly Applications

**DOI:** 10.1155/2013/716545

**Published:** 2013-11-17

**Authors:** Shamkant B. Badgujar, Raghunath T. Mahajan

**Affiliations:** ^1^Department of Biochemistry, National Institute for Research in Reproductive Health (ICMR), Jehangir Merwanji Street, Parel, Mumbai, Maharashtra 400 012, India; ^2^Faculty of Science, Department of Biotechnology, Moolji Jaitha College, North Maharashtra University, Jalgaon, Maharashtra 425002, India

## Abstract

An antigenic glycosylated cysteine protease has been purified from the latex of *Euphorbia nivulia* Buch.-Ham. It exhibits remarkable protease activity in the presence of metal ions, oxidizing agents, organic solvents, and detergents. This enzyme showed potential role in leather processing industry due to its dehairing activity for animal hide without hydrolyzing fibrous proteins, producing, by this way, a better quality product. The enzyme can also be used for silver recovering from X-ray plates. In addition, the stability (temperature and surfactants) and hydrolysis of blood stain data also revealed its application in detergent industries. Agriculturally, this protease finds application in biocontrol process against the infectious management of root knot nematode, *Meloidogyne incognita*. Biologically, it shows noticeable wound healing, haemostatic and antibacterial activity.

## 1. Introduction

Latex is a milky fluid composed of a liquid serum holding in suspension or in solution, a complex mixture of constituents. It may contain a variety of cellular components, like nuclei, mitochondria, ribosome-like particles, and lysosome analogues. Agglomerative low density materials, such as various enzymes, terpenes, alkaloids, vitamins, carbohydrates, lipids, and free amino acids have been identified among the components [1]. The characteristic feature of plant from *Euphorbiaceae*, *Apocynaceae*, *Moraceae*, *Asclepiadaceae*, *Sapotaceae*, *Caricaceae,* and *Convolvulaceae* families has latex secreting properties. Latex has been reported to occur in 12000 plant species belonging to 900 genera. A number of proteases from latex bearing species have been isolated and characterized [2]. Additionally, in our laboratory, we have studied proteolytic activities of 21 latex bearing plants belonging to seven different laticiferous families. A common feature that can be found in the latex of the Euphorbiaceae is the presence of noticeable proteolytic activity [3]. *Euphorbia* is a large genus consisting of about 2000 species. About 52 species have been recorded from India. The genus includes herbs, shrubs, and trees of widely diverse habitats [4, 5]. The present study was carried out on the proteolytic activity of latex of *Euphorbia nivulia *Buch.-Ham. This is a wild, thorny, xerophytic, succulent plant, found in boundaries of the agricultural field and also in dry barren areas. The secretion of milky juice is a characteristic property of this plant. Phytochemical studies have led to the isolation of ingol diterpenes (3-acetyl-8-methoxyl-7-angolyl-12-hydroxylingol; 3,12-diacetyl-7-hydroxy-8-methoxylingol; 3,12-diacetyl-7-angolyl-8-hydroxylingol; 3,12-diacetyl-8-benzoylingol; and 3,12-diacetyl-7-benzoyl-8-nicotinylingol) along with three macrocyclic ingol diterpenes derivatives (3,7,12-triacetyl-8-benzoylingol; 3,12-diacetyl-7-angeloyl-8-methoxyingol; and 7-angeloyl-12-acetyl-8-methoxyingol) [6]. The latex of *E. nivulia* has been cited for its antioxidant, immunomodulator, cytotoxic, anti-inflammatory, wound healing, haemostatic, and antiproliferative activity [5]. During the course of screening for biochemical constituents, a substantial amount of proteolytic and milk clotting activity was found in the latex of this plant [7]. Recently, an attempt has been made on peptide sequencing of 31 kDa, Tubulin alpha-1 chain-like protein called Nivulian-I, present in the latex of *E. nivulia *[6]. Very recently, a comparative account on proteolytic activity of *E. nivulia* and other three plants, namely, *Calotropis procera*, *Carica papaya,* and *Ficus carica,* was reported by us. Additionally, we report the glycosylated cysteine protease called Nivulian-II of the latex of *E. nivulia *[6]. This paper describes the further biochemical characterization of this cysteine-like protease with some ecofriendly applications.

## 2. Materials and Methods

### 2.1. Chemicals

All chemicals with the highest purity, analytical HPLC grade were purchased from Sigma Chemicals, USA; Himedia Laboratories, Mumbai; SRL Chemicals, Mumbai; Qualigen Fine Chemicals, Mumbai; Merck Chemicals, India; and Bangalore Genie, India.

### 2.2. Animals Used

Four- to five-week-old albino Wister male rats (120 to 180 g body weight) were used for antigenicity. Swiss albino mice of either sex (50–100 g) were used for the study of wound healing activity. The animals were maintained under standard laboratory conditions in animal house approved by Committee for the Purpose of Control and Supervision on Experiments on Animals (CPCSEA, registration number 1062/C/07/CPCSEA/24, May 2007). The experimental protocol was approved by Institutional Animal Ethics Committee constituted under CPCSEA rules, India. 

### 2.3. Animal Hides

Freshly flayed wet rat hide was obtained from animal house of Moolji Jaitha College, North Maharashtra University, Jalgaon, India. Cow hide of healthy animal was collected from local meat shops of Jalgaon city.

### 2.4. Blood Sample

Fresh blood samples of domestic animals namely, goat (*Capra hircus*), buffalo (*Bubalus bubalis*), and ox (*Ovibos moschatus*), of either sexes were collected under the supervision of Dr. N. M. Pawar, veterinary practitioner of Paldhi unit, Jalgaon District, Maharashtra, India. Blood sample of healthy hen was collected from local chicken shops of Jalgaon city (M.S.).

### 2.5. Nematode Sample

Root-knot nematodes (*Meloidogyne incognita*) were collected along with soil sample by zig-zag manner from vicinity of Department of Agricultural Entomology, Mahatma Phule Krishi Vidyapeeth, Rahuri Ahmednagar, Maharashtra, India.

### 2.6. Microbial Culture

The microbial cultures like *Staphylococcus aureus *(ATCC 25923), *Escherichia coli* (ATCC 25992), *Klebsiella pneumoniae *(ATCC 23357), *Pseudomonas aeruginosa *(ATCC 27853), *Proteus vulgaris *(NCIM 2027), and *Bacillus subtilis *(NCIM 2063) were procured from National Collection of Industrial Microorganisms (NCIM), Pune, Maharashtra, India.

### 2.7. Plant Material, Collection of Latex, Preparation of Crude Enzyme, and Purification of Cysteine Protease

The detailed information about identification, collection, preservation, and preparation of crude enzyme and its proteolytic activity of *Euphorbia nivulia* latex and its quantification is described in our previous communication [6]. Method of purification of protease was done using acetone precipitation, DEAE cellulose chromatography, and dialysis and followed by rechromatography on DEAE cellulose column as described in our earlier communication [8].

### 2.8. Characterization of Cysteine Protease 

#### 2.8.1. Thermal Stability

The thermal behavior of the purified enzyme fraction was evaluated by incubating the enzyme at the desired temperatures, in the range of 20–800°C for 15 min in 0.01 M phosphate buffer (pH 6.6), and an aliquot was used for the enzyme activity measurement at the same temperature. At each temperature, a control assay was carried out without enzyme.

#### 2.8.2. Effect of Metal Ions on Proteolytic Activity

Impact of various metal ions, namely, K^+^, Na^+^, Zn^++^, Ag^++^, Cd^++^, Fe^++^, Hg^++^, Mg^++^, and Mn^++^, at 5 mM concentration on the enzyme catalytic behaviour was studied. The enzyme fraction along with 0.01 M phosphate buffer (at optimum pH) was preincubated at room temperature for 30 minutes with respective metal ions separately before enzyme assay. Then, the residual proteolytic activity was measured by using casein according to standard assay procedure relative to control (without metal ion).

#### 2.8.3. Effect of Surfactant and Oxidizing Agent on Proteolytic Activity

The effect of different surfactants, namely, sodium dodecyl sulphate, Triton X-100, and Tween 80, and oxidizing agent (H_2_O_2_) at 1% (v/v) final concentration on enzyme activity was studied by preincubating enzyme preparation for 60 min at 37°C in the above surfactants and oxidizing agent before analysis. Then, the residual proteolytic activity was measured by using casein according to standard assay procedure relative to control (without chemical surfactants and oxidizing agent) and the resulting activity was taken as 100 percent.

#### 2.8.4. Effect of Organic Solvents on Proteolytic Activity

The stability of enzyme activity in presence of different water miscible or immiscible organic solvents was investigated by incubating the enzyme preparation with various organic solvents (30, 50, and 70%, v/v) at 37°C for 1 h. After incubation, the residual enzyme activity was determined as per the previously discussed method with casein. The enzyme activity of a control sample (without solvent), incubated under the same conditions, was taken as 100 percent [9]. For this assay, the solvents used were acetone, acetophenone, benzyl alcohol, benzene, butanol, chloroform, chlorobenzene, dichloromethane, dimethylformamide, diethyl ether, ethylene glycol, ethyl acetate, methanol, propanol, tetrahydrofuran, and trichloroethylene (Merc Chemicals). 

#### 2.8.5. Compatibility of Protease with Laundry Detergents

The compatibility of enzyme preparation in presence of commercial solid laundry detergents was examined by incubating enzyme preparation for 60 min at 37°C with various detergent preparations, and the residual enzyme activity was determined as per the method using casein. The enzyme activity of a control sample (without detergent), incubated under the same conditions, was taken as 100 percent. The solid detergents used were Ariel Oxy Blue, Tide (Procter and Gamble Company, USA), Fena Ultra, New Impact (Fena (P) Ltd., New Delhi, India), Nirma (Nirma Ltd., Ahmedabad, India), Wheel Active, Rin, Surf Excel (Hindustan Liver Ltd., India), and Ujala (Jyoti Laboratories Ltd., Mumbai, India). The detergents were diluted in tap water to give a final concentration of 7 mg/mL and 10 mg/mL to simulate washing conditions. The endogenous proteases contained in these detergents were inactivated by incubating the diluted detergents at 65°C for 60 min, prior to the addition of enzyme [10].

### 2.9. Antigenic Property

#### 2.9.1. Immunization of Rat and Production of Polyclonal Antisera

The protein sample (100 *μ*g in 0.05 mL of phosphate buffer saline; 0.01 M, pH 6.0), thoroughly mixed with equal volume of Freund's complete adjuvant and injected into male albino Wister rat (180 to 200 g body weight) subcutaneously at multiple sites. Two booster doses were administered at weekly intervals with the same concentration but with equal volume of Freund's incomplete adjuvant. After nine days from last booster dose, blood was drawn through retroorbital plexus/sinus with a glass capillary tube, and antisera was separated after allowing the blood to coagulate at 8°C for 24 h.

#### 2.9.2. Detection of Antibodies and Immunological Cross Reactivity

The presence of antibodies was confirmed by Ouchterlony's double immunodiffusion assay [11]. 1% agarose in phosphate buffer saline containing 0.02% sodium azide was solidified on glass plate, and appropriate holes (5 mm diameter) were punched into it. The desired concentration of antigen (100 *μ*g) of protein sample, that is, enzyme preparation in peripheral wells, and 20 *μ*L of antiprotein serum were loaded in the side wells and left at 28°C for 24 to 48 h for visualization of precipitin line. Then, the antigen antibody reaction (precipitin line) was verified simultaneously by loading preimmune serum (considered as control).

### 2.10. Ecofriendly Applications of Cysteine Protease

#### 2.10.1. Dehairing Studies

The fresh fleshed rat and cow hide were washed with a commercial detergent and cut into 5 × 5 cm pieces. Eight to twelve grams of hide (usually two to three pieces) was processed in a flask with crude cysteine protease (CCP) and 0.01 M phosphate buffer (control) in a proportion of 5.0 mL of liquid enzyme per g of hide. At the end of the process, the hide pieces were gently scraped with fingers to remove loose hairs. This procedure was necessary because rubbing in this laboratory-scale process was not as vigorous as in industrial drums. The skin depilation started with 5.0 U/mg protein concentrations of enzyme, and it was completed during 18 h at 7.0 pH/30°C temperature. 

#### 2.10.2. Histochemical Studies of Dehaired Skin

The tissue samples of protease-treated hides were put in 10% formol saline. The treated samples were processed in ascending grades of alcohol, cleaned in xylene, and then embedded in the paraffin wax for making tissue blocks. Sections of 4 mm (5 *μ*m thick) tissue were obtained using microtome after embedding in paraffin wax block, and they were stained using Hematoxylin and Eosin to examine the histological features as per protocol [12, 13].

#### 2.10.3. Evaluation of Washing Performance of Crude Cysteine Protease

Application of protease (5 U mg protein^−1^) in 0.02 mol L^−1^ phosphate buffer pH 7.4 as a detergent additive was studied on white cotton cloth pieces (1.5′′ × 1.5′′) stained with blood samples of different animals (ox, buffalo, goat, hen, and human being). The stained cloth pieces were taken in separate trays. The following groups were setup: (A) tray with 50 mL of 0.02 mol L^−1^ phosphate buffer pH 7.4 and blood stained cloth; (B) tray with 50 mL protease (5 U mg protein^−1^) in 0.02 mol L^−1^ phosphate buffer pH 7.4 and blood stained cloth; (C) Tray with 50 mL detergent (7 mg/mL) and blood stained cloth. (D) tray with 50 mL mixture of detergent (7 mg/mL), protease (5 U mg protein^−1^), and blood stained cloth [14]. These trays were incubated at 30°C for 25 min. The cloth pieces were taken out from each set at regular intervals of 5 min, rinsed with water, dried, and visually examined. Untreated cloth pieces stained with blood were taken as control. Additionally, after washing performance, dried cotton pieces were subjected to cutting. The resulting small pieces of individual destained cotton cloth piece were suspended in normal saline at 30°C and centrifuged at 5000 rpm for 20 min. The progress of destaining of blood stain was monitored by measuring the absorbance of resulting supernatant at 420 nm. The test cotton fabric pieces stained with egg yolk were also treated under similar conditions at 30°C. Stain removal was checked qualitatively by visualization. 

#### 2.10.4. Hydrolysis of Gelatin and Release of Silver

Used X-ray films were washed with distilled water and wiped with cotton impregnated with ethanol. The washed film was dried at room temperature for 30 min. One g of X-ray film (cut into 2 × 2 cm pieces) was then incubated with 10 mL of protease (5 U mg protein^−1^) in 0.02 mol L^−1^ phosphate buffer pH 7.4 (such that the film is completely immersed in the enzyme) at 30°C with continuous shaking. Turbidity of the reaction mixture (hydrolysate) increased with time (as the hydrolysis progressed), and no further increase in turbidity was observed when hydrolysis was completed. Samples were removed at 30 min intervals, and time required for complete removal of gelatin layer was noted [15].

### 2.11. Biological Activities of Cysteine Protease

#### 2.11.1. Wound Healing Activity (Excision Wound Model)

Albino mice were divided into three groups of six animals in each group. Circular wounds of approximately 300 to 350 mm^2^ in diameter were inflicted on the shaved skin under mild ether anesthesia. Groups II and III were treated with crude cysteine protease (CCP) and 1% w/w framycetin sulphate I.P (Soframycin), respectively. Group I was untreated and considered as control. The progressive changes in wound area were recorded in mm^2^ by tracing the wound boundaries around it on a transparent paper on every day. Wound contraction was expressed as percentage reduction of original wound size [16].

#### 2.11.2. Coagulation Time of Whole Blood

Twelve tubes were arranged in a water bath at 37°C. Into six of these tubes (test), 0.1 mL of the crude cysteine protease (CCP) was added, and nothing was added to the remaining six tubes (control). 0.5 mL of blood was collected separately from mice by clean venipuncture, and 0.5 mL was added into each of the tubes; the tubes were observed for clot formation, and the clotting time was recorded using a stop watch. The average of the clotting time of the six tubes with protease (test) and the six tubes without protease (control) were taken as the clotting time, respectively [17].

#### 2.11.3. Bleeding/Clotting Time Test

The effect of the protease on bleeding from fresh experimentally induced wounds was evaluated using the bleeding/clotting time test in mice [18]. After sterilizing the skin with 70% alcohol, a puncture was made on the tail with a sterile sharp blade. Immediately, a drop of the CCP (1000 *μ*g/mL) was placed on the cut portion, and at the same time a stopwatch was switched on. Sterilized filter paper was used to absorb blood coming out, and time taken for ceasing bleeding was recorded; the average was taken as bleeding time (test). The procedure was repeated on the second group of mice, but here, after puncturing the tail, a drop of protease (CCP) was not applied, serving it as a control group of animal. 

#### 2.11.4. Antimicrobial Activity

Antimicrobial activity of different concentrations of CCP (50 and 100 *μ*g/mL) was studied against Gram positive and Gram negative bacteria by using disc diffusion method [19]. After overnight incubation at 37°C, the zone of inhibition was measured and compared with reference antibiotic (Gentamicin). A control experiment was set up by using an equal amount of phosphate buffer in place of protease. 

### 2.12. Agricultural Application: Nematicidal Activity

The *Bhendi *(Okra) crops were grouped into eight groups with six plants in each group.

*Group I.* Normal sterile soil plant (control or untreated-uninoculated).

*Group II.* Nematode affected plant (untreated).

*Groups III, IV, V, and VI.* Nematode control by 2.5, 5.0, 7.5, and 10.0 mg of CCP per g soil, respectively. 

*Groups VII and VIII.* Nematode control by carbofuran 3% G nematicide and Sanjeevani 1% WP (*Trichoderma viride*), respectively: dose 2.0 mg per g soil. 

The study was undertaken in 48 earthen pots (15 cm diameter). Each was filled with 800 g of sterile soil along with Bio Organic Fertilizer (1 : 1). Experimental okra seed was grown in each and every pot under greenhouse condition. After 15 days of germination, the inoculation was achieved by pouring the nematode water suspension containing 2000 nematodes through 4 holes (5–8 cm depth) around the root system of experimental crop, which were immediately covered by sterile soil, except Group I pots. Exactly after 15 days, CCP was applied in four doses, that is, 2.5, 5.0, 7.5 and 10.0 mg per g soil (Groups III, IV, V, and VI). Carbofuran 3% G (encapsulated) nematicide (Group VII) and Sanjeevani 1% WP (*Trichoderma viride*) (Group VIII) were also applied separately in 2.0 mg per g soil by the same procedure described earlier. Then exactly after 20 days, we counted the number of nematodes to determine the percent mortality, that is, PM (PM = (Number of dead nematodes/Total number of nematodes) × 100) [20].

## 3. Results and Discussion

### 3.1. Characterization of Cysteine Protease

*Euphorbia nivulia* Buch.-Ham. belongs to the Euphorbiaceae family, whose members are characterized by secretory tissues (laticifers) which frequently include proteolytic and milk clotting enzymes. The young stem latex of *E. nivulia* possesses proteolytic and milk clotting enzyme in more quantity as compared with other investigated laticiferous plants of northern region of Maharashtra, India [6]. *E. nivulia* latex contains a thermostable glycosylated cysteine protease presenting an optimum activity at pH of 6.6 and temperature of 45°C, a molecular weight of 43.42 kDa (analyzed by SDDS-PAGE), is activated by cysteine hydrochloride and inhibited by mercuric chloride [6]. According to protease nomenclature, this protein is designated as Nivulian-II due to previously Nivulian-I (31486.985 Da) was already characterized from *E. nivulia* latex [21]. 

**Figure 1:**
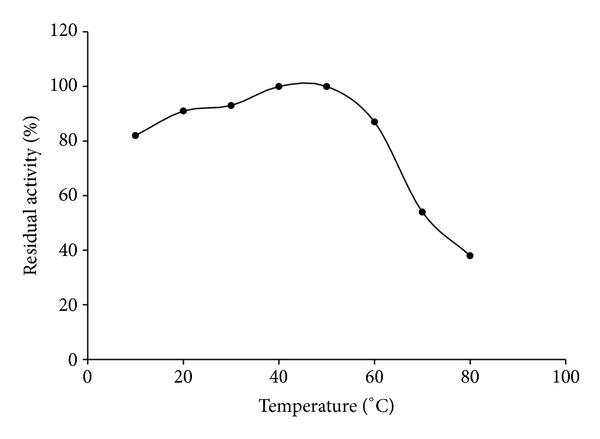
Thermal stability of protease.

The stability of proteins and enzymes is usually a factor that limits their usefulness in many applications. The thermal stability of this cysteine protease was examined by measuring the residual enzyme activity of aliquots of the enzyme by incubating at different temperatures. This cysteine protease was stable up to 60°C. At least, 80% residual proteolytic activity of cysteine protease was retained after incubation at 60°C as shown in Figure 1. After enzyme incubation at 60 and 70°C, the enzyme activity was retained about 87.43 and 54.36 percent activity, respectively. The enzyme was almost completely inactivated by heating for 15 min at 80°C. Similar thermal profile of cysteine protease was reported in cysteine protease of *Araujia hortorum *fruits, freesia corms, and endopeptidase of *Bromelia hieronymi* [22–24].

The effect of various metal ions at 5 mmol lit^−1^ concentration on the proteolytic activity at 37°C is summarized in Table 1. Enzyme activity was affected by all the examined metal cations except Ca^++^, Mg^++^, and Mn^++^. Enzyme activity was affected by (i) 25–35% in presence of Fe^++^ and Na^++^ ions, (ii) 40–46% in presence of K^+^, Zn^++^, Ag^++^, and Cd^++^ ions, and (iii) 89–92% in presence of Hg^++^ ion. A similar inhibition profile was also reported with characterization of a cysteine protease isolated from wheat grain *Triticum aestivum *[25]. The effect of surfactants and oxidizing agent at 1% concentration on the proteolytic activity at 37°C is also summarized in the same table. In presence of SDS and H_2_O_2_, protease activity was inhibited up to 48.49% and 31.82%, respectively, and activity was unaffected by exposure to Triton X-100 and Tween-80, indicating that the purified protease could not be lipoprotein. Our results are in good agreement with the earlier observations reported in the purification of metalloprotease of *Pseudomonas aeruginosa *[26].

Enzymes are generally inactivated in the presence of organic solvents. The partially purified protease retained its 85 to 100% activity at three concentrations of organic solvents, that is, (i) 30% solvents of acetone, benzene, dimethylformamide, ethyleneglycol, and propanol, (ii) 50% solvents of acetone, acetophenone, benzene, chlorobenzene, and dimethylformamide, and (iii) 70% solvents of acetone and benzene, but more than 60% inactivation was observed in presence of remaining solvents (Table 2). Amongst the organic solvents studied, dichloromethane and butanol strongly inhibited protease activity. Acetone causes slightly increased protease activity. The enzyme was stable in the presence of acetone, benzene and ethyleneglycol. Our results are in good agreement with earlier observations [27]. 

The high activity and stability of the enzyme preparation in the pH range from 5.0 to 8.0, and its relative stability towards surfactants is very useful for its application as detergent additive. To check the compatibility of the enzyme preparation with commercial solid detergents, the enzyme was preincubated in the presence of various commercial laundry detergents for 60 min at 37°C. The data presented in Table 3 illustrates that the enzyme is extremely stable in the presence of detergents like Tide, Ujala, and Nirma at both concentration of 7 mg/mL and 10 mg/mL. The enzyme retained 90 to 98% of its activity in the presence of Wheel and Ariel and 80 to 90% in presence of Rin, Surf Excel, Fenna, and Ghari at 7 mg/mL. Enzyme preparation was less stable in presence of Sasa and Impact, where it lost up to 22 to 33% of its activity. The obtained results clearly indicate that the performance of enzyme in detergents depends on a number of factors, including the detergents' compounds since the proteolytic stability varied with each laundry detergent. The mentioned compatibility of the enzyme preparation with commercial solid detergents was in accordance with previous studies [10, 13, 27].

**Figure 2:**
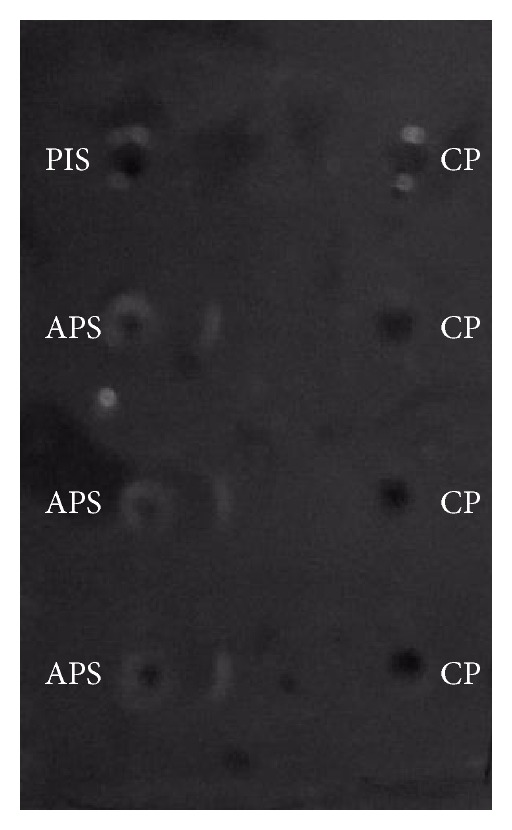
Ouchterlony's double immunodiffusion. PIS: preimmune serum; APS: antiprotein serum (Anticysteine protease serum); and CP: cysteine protease.

Polyclonal antibodies against protein (cysteine protease) were raised in male albino Wister rats. The presence of antibodies in the anti rat serum was checked by Ouchterlony's double immunodiffusion method. Precipitin lines start appearing after 10–12 h of incubation at 28°C and are distinctly visible in about 36 to 42 h (Figure 2). Additionally, the polyclonal antibodies are specific to specific concentration of glycoprotein. This observation confirms that the proteins present in the enzyme fractions are distinct. It reveals that the glycoprotein has unique antigenic determinants. The polyclonal antibodies raised for glycosylated cysteine protease would be of immense importance in detecting and as a ligand for various future studies. Our results are in good agreement with earlier observations of antigenic property of cysteine protease named Procerain of *Calotropis procera *latex [28], Milin, a protease of *Euphorbia milii *latex [29], Cryptolepain of *Cryptolepis buchanani *latex [30], Indicain, latex protease of *Morus indica *[31], and a novel cysteine protease named Procerain B of *Calotropis procera* latex [32].

### 3.2. Ecofriendly Applications of Cysteine Protease

**Figure 3:**
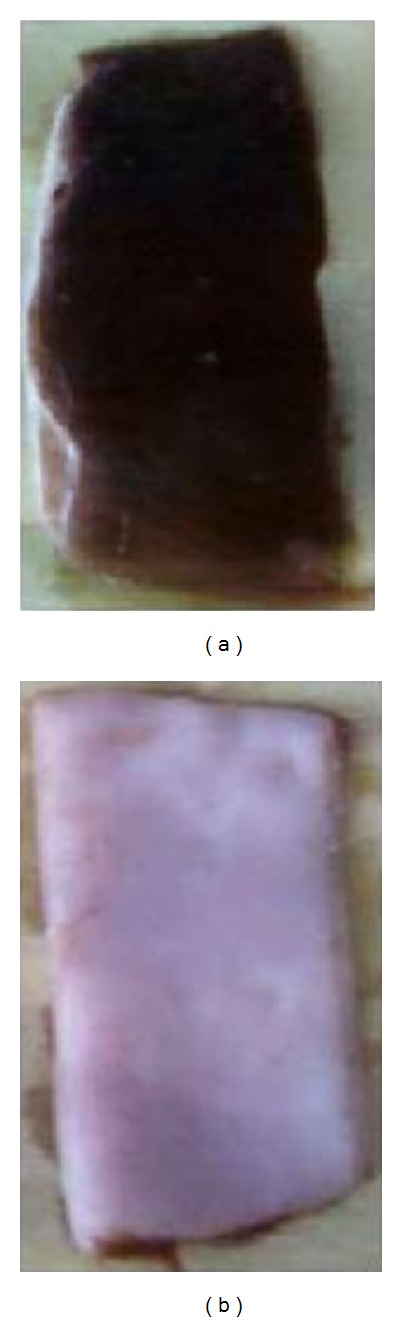
Dehairing of cow hide. (a) Cow hide control and (b) cow hide after 18 h incubation with CCP.

**Figure 4:**
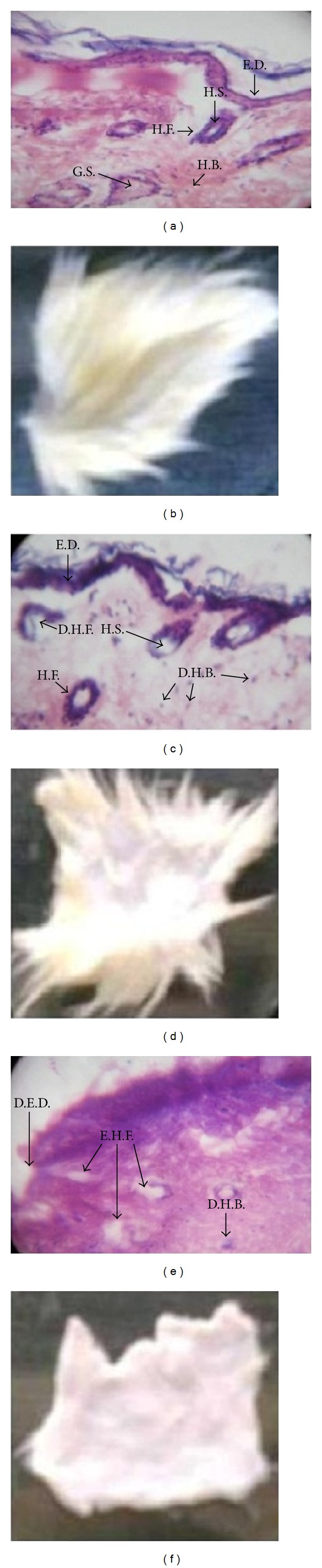
Dehairing of rat hide. Hematoxylin and eosin staining skin sections from (a) control without treatment; (c) partially dehaired pelts of enzymatic process after 12 h, and (e) dehaired pelts of enzymatic process after 18 h incubation with CCP. Rat hide images from (b) control without treatment; (d) after 12 h incubation with CCP; and (f) after 18 h incubation with CCP. E.D.: epidermis; H.S.: hair shaft; H.F.: hair follicles; G.S.: glandular structure; H.B.: hair bulb; D.H.B.: degraded hair bulb; D.E.D.: degraded epidermis; and E.H.F.: empty hair follicles. D.H.F.: degraded hair follicles.

Enzymatic dehairing process is gaining importance as an alternative chemical methodology in present day scenario. This process is significant in reduction of toxicity in addition to improvement of leather quality [33]. The experimental dehaired pelts of cow and rat hide showed complete removal of fine hairs (Figures 3 and 4) with increased brightness, and it may be due to elimination of sulfide in the process. Similar results are noticed by Subba Rao et al. [13] with thermostable protease of *Bacillus circulans*. Cysteine protease could remove hairs of rat and cow hides after 18 h incubation with hide at 30°C easily compared to each control, with no observable damage on the collagen. Therefore, the dehaired skin exhibits clean hair pore and clear grain structure.

Histological sections of dehaired pelts stained with hematoxylin and eosin revealed the removal of epidermis, glandular structures, hair shafts, bulb, and follicles (Figures 4(c) and 4(e)). Complete absence of the previous structural features along with opening up of collagen fibre structure was seen with samples (hide) treated for more than 12 h (Figure 4(e)). On the other hand, partial and moderate removal of hair is observed with 12 h incubation (Figure 4(c)). The data depicted that there was no apparent damage to the collagen fibres in dehaired pelts (Figure 4(e)). The histological studies further strengthened the easy removal as the hair follicles were found to be empty (Figure 4(e)). The skin with no protease treatment showed hair follicles in the dermis with intact hair (Figure 4(a)), and hair was also observed coming out of the epidermal layer. This showed that the protease was effective in removing hair from hair follicles. Comparing treated skins with untreated controls, we observed that only skin epidermis and adnexa (skin appendages), including hair bulbs, were digested, showing a histological autolytic-like appearance (Figures 4(c) and 4(e)), as could be expected for proper skin depilation. Protease enzyme has advantages in dehairing process as this enzyme effectively unhaired the rat and cow hides within 18 h, compared to earlier reports where proteases from *Bacillus cereus* and *Aspergillus tamarii* dehaired the goat skin in 21 and 24 h, respectively, [33, 34] indicating its potential application in leather industry for ecofriendly economizing the process. 

**Figure 5:**
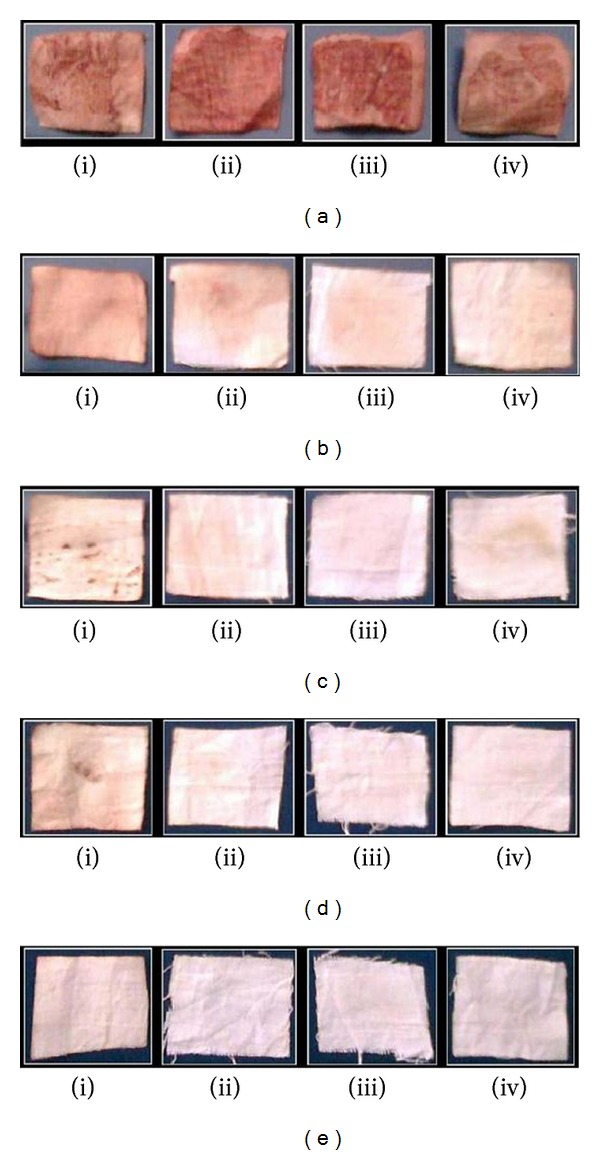
Evaluation of protease (CCP) for washing of goat's blood stains from cloth (a) Control; (b) 5 min treated; (c) 10 min treated; (d) 15 min treated; (e) 25 min treated; (i) Buffer treated; (ii) Detergent treated; (iii) CCP treated and (iv) CCP + Detergent treated group.

The results (Figure 5) of evaluation of enzyme for washing performance pointed out that the blood stains on the cloth pieces remained as they were even after 15 min of rinsing in the case of controls and commercial detergents. Blood stain was completely removed from the cloths after rinsing them with a combination of detergent and crude cysteine protease (CCP) for a period of 15 min, whereas it was removed after 25 min when rinsed with CCP individually. These results clearly indicate that the enzyme is fairly stable as an ingredient in the presence of detergents. Our results of washing performance of CCP are in good accordance with the earlier observations reported in washing performance of protease of *Pseudomonas aeruginosa* [35] and *Streptomyces gulbdrgensis* [14].

**Figure 6:**
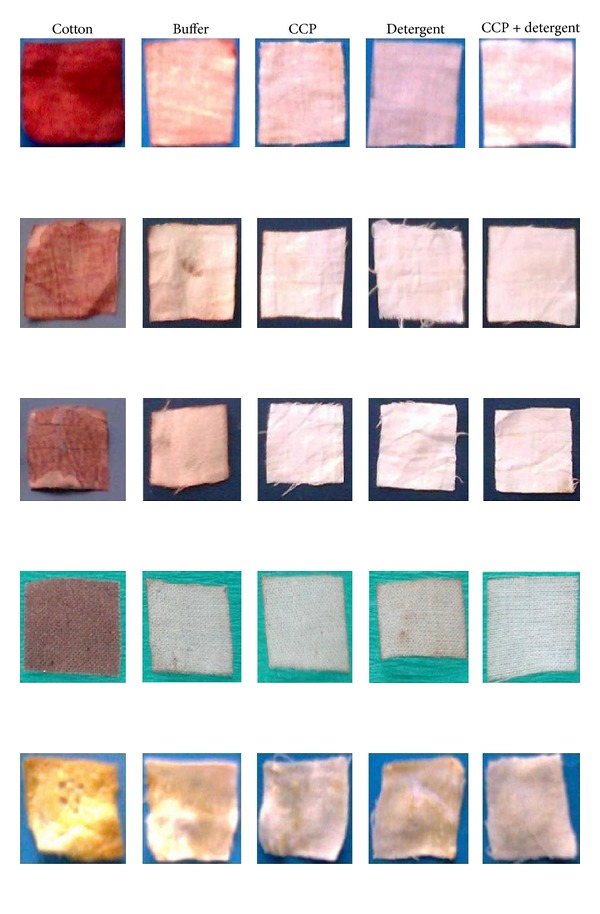
Removing blood and egg yolk stains from cloth by the application of protease (CCP) and detergent after 25 min. First column: Untreated blood stained cloth (control); second column: blood stained cloth washed (BSCW) by buffer; third column: BSCW by CCP; fourth column BSCW by detergent; fifth column: BSCW by detergent with CCP. First, second, third, and fourth row: cloth stained by blood sample of human being, ox, buffalo, and hen, respectively. Fifth row: cloth stained by egg yolk.

In order to evaluate the performance of CCP with respect to its capability of removing stains of different blood samples, namely, human being, ox, buffalo, and hen, egg yolk stains were used. On incubating several pieces of stained cloth at 30°C for 25 min, results of these findings are interesting; the use of enzyme alone showed more effective removal of blood and egg yolk stains (Figure 6). In fact, protease facilitates the release of proteinaceous materials in a much easier way than the commercially available detergent. Furthermore, the combination of CCP with detergent resulted in complete stain removal (Figure 6). A similar study reported on the usefulness of alkaline proteases from *Bacillus brevis* [36] and *Bacillus pumilus* [37]. These destaining profiles made clear the idea about removal of blood stains with minimum use of commercial detergent within 25 min. Rapid blood stain removal was noticed with supplementation of commercially available detergents (Table 4). Similar results of destaining of blood with combination of protease and detergent were noticed by Subba Rao et al. [13].

**Figure 7:**
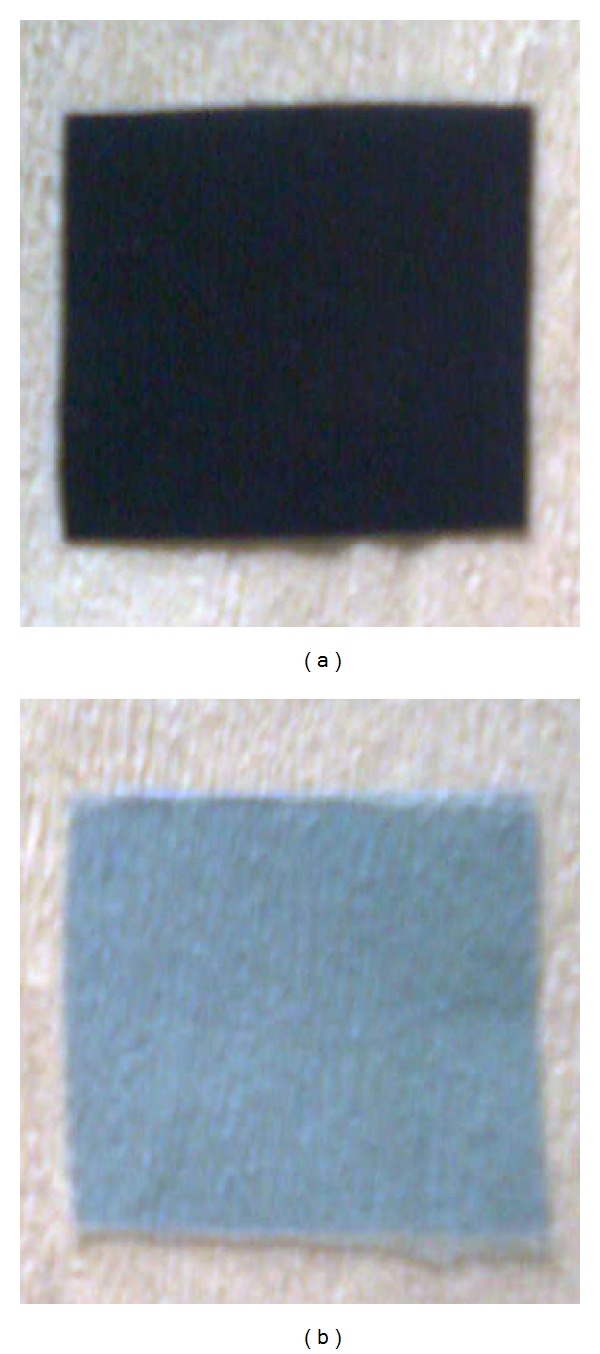
Decomposition of gelatin layer of X-ray film by protease. (a) Control; (b) experimental: X-ray film after 3 h exposure to protease.

Treatment of X-ray films with protease resulted in the sliver bound with gelatin being stripped off into the reaction mixture, and this results in clean appearance of plastic film. The loss in weight after the treatment was around 5% (w/w) based on initial weight of the film (1.0 g). The gelatin layer of the X-ray film was completely removed after 3 h of enzymatic exposure as compared with that of the control (Figure 7). Earlier studies reported similar observations [26] in bacterial species. 

### 3.3. Biological Activities of Cysteine Protease

The tropical application CCP significantly reduces the bleeding and clotting time in mice up to 8 and 14 seconds, respectively, as compared with control values which were found to be 57 and 60 seconds. Also, the cysteine protease significantly decreased the coagulation time of whole blood in mice (*P* < 0.001). A significantly improved wound healing activity has been observed in mice treated by cysteine protease as compared to that of reference standard (Soframycin) and control groups of animal (Table 5). The study reveals that, in all three groups of animal, wound area was reduced progressively. However, on 16th postwounding day, Group I animals have 92.02% wound contraction (which may be due to self-immunity of the animals), whereas in Groups II and III animals exhibit 93.43 and 93.29% effect by Soframycin and CCP, respectively. An enzyme treated group of animals showed significant reduction in wound contraction area (*P* < 0.001). A similar type of wound healing activity was reported in few Euphorbiaceae members, that is, *Jatropha curcas *[38] and *Euphorbia neriifolia* [39], and a member of Asclepiadaceae family, that is, *Calotropis procera* [40]. Our results agreed with the findings of earlier researchers. Present result fully justifies the folkloric use of *E. nivulia *for healing wound [41, 42]. Evaluation of the potentials of *E. nivulia *in wound management showed that the cysteine protease of plant latex has haemostatic and wound healing properties. The protease arrested bleeding from fresh wounds by reducing bleeding/clotting and whole blood coagulation time which are important indices of haemostatic activity. Our results are in good agreement with the earlier observations of haemostatic activity of stem latex of another member of Euphorbiaceae family, that is, *Jatropha gossypifolia* [43]. The reduction in coagulation time of whole blood by the protease (CCP) indicates that it may also interfere with the blood coagulation pathways. Thus, this shrub is a promising haemostatic agent and wound healing promoter.

The results obtained from the disc diffusion assay (Table 6) showed that there was an increasing effect on bacterial growth inhibition with increasing concentration of protease (CCP). It showed comparable inhibitory activity on almost all the bacteria tested. Protease had a strong antibacterial activity against *Escherichia coli* and *Staphylococcus aureus, *moderate against* Pseudomonas aeruginosa, *and lowest activity against *Klebsiella pneumoniae*, *Proteus vulgaris, *and *Bacillus subtilis*. The result concludes that the protease has exhibited a potent antibacterial activity. 

The final population of *Meloidogyne incognita* in untreated pots and pots treated with CCP (10 mg/g), *Trichoderma viride *(Sanjeevani) and carbofuran (standard nematicide), was 3800, 227, 85, and 160 per pot, respectively, as against the initial population of 2000 (Table 7). The percent mortality of nematode was raised with increasing concentration of protease (CCP). The moderate dose of protease (5 mg) was equivalent to the 1 mg of standard nematicide.

 The results in Table 8 summarize the morphological observation of experimental plants (dose level of CCP is 10 mg/g of rhizosphere soil) and standard nematicides (2 mg/g of soil) in the form of seed index, whole plant height, spad value, and dry biomass of okra plants. Seed index is the ratio of weight of seed to weight of fresh fruit. Highest value of seed index was found in the CCP exposing crop plant, that is, 0.0015, followed by Sanjeevani exposing crop plant, while in nematode infected plant and control it was recorded as 0.005 and 0.008, respectively. The weight of root (g) for the corresponding treatment was 0.863, 1.032, and 0.872 (carbofuran exposing plant), respectively, as against 0.640 for untreated-uninoculated control. Spad value indicates the intensity of chlorophyll contents. The lower the spad value the less the chlorophyll intensity or vice versa. Chlorophyll content of leaves of experimental plant was measured digitally in terms of spad value with the help of spad chlorophyll meter or as simply called Spadometer, which is a small electronic punching machine with chlorophyll detecting sensor [44]. Spad values for the treatment of carbofuran, Sanjeevani, and protease were 33.04, 34.09, and 30.12, respectively, as compared to 37.85 for the control, while whole plant height was 51.95, 55.20 and 57.15, respectively, as compared to 58.02 for the control.

The results showed that the reproduction of *Meloidogyne incognita *in plants grown in soil treated with protease of plant latex was significantly suppressed. Protease treated plants showed more significant increase in the whole plant height than that of untreated-uninoculated soil plant. However, nematode population is declined only in pots treated with protease and standard nematicide, whereas, in untreated-inoculated pots it has almost doubled. It is, therefore, reasonable to believe that plants grown with protease develop certain degree of resistance against nematode attack. It may be due to the absorption of substances liberated during decomposition of protein by soil microbial flora [45]. 

In conclusion, characterization and environmental friendly potential application of antigenic glycosylated stable cysteine protease of *Euphorbia nivulia *latex were studied for the first time. The enzyme revealed excellent stability and compatibility towards temperature, metal ions, detergents, oxidizing agents, surfactants, and organic solvents. Studies indicated its utility for blood stain removal and detergent and dehairing properties. The physical properties of the experimental rat's pelt revealed effective dehairing of fine hairs completely within 18 h without sodium sulfate indicating its ecofriendly nature in dehairing. Hematoxylin and eosin staining revealed the removal of epidermis, glandular structures, hair shafts, and follicles with complete opening of collagen fiber structure. Medicinally, it shows noticeable wound healing and haemostatic activity. Agriculturally, it finds application in biocontrol process against the infectious management of root knot nematode.

## Figures and Tables

**Table 1:** Effect of surfactants, oxidizing agent, and metal ions on proteolytic activity.

Metal ions/O.A./Sur.	Concentration	Residual activity (%)
Control	Nil	100
SDS	1%	51.51 ± 0.18
H_2_O_2_	1%	68.18 ± 0.35
Tween 80	1%	100 ± 0.69
Triton X-100	1%	100 ± 1.04
KCl	5 mM	59.64 ± 0.09
NaCl	5 mM	64.97 ± 0.20
ZnCl_2_	5 mM	58.46 ± 0.16
AgSO_4_	5 mM	56.69 ± 0.04
CaCl_2_	5 mM	93.20 ± 0.16
CdCl_2_	5 mM	53.50 ± 0.29
HgCl_2_	5 mM	09.43 ± 0.57
FeCl_3_	5 mM	73.50 ± 0.17
MgCl_2_	5 mM	97.01 ± 0.22
MnCl_2_	5 mM	99.89 ± 0.45

Data represented in average values ± SD of *n* = 6 experiment, O.A.: oxidizing agent; Sur: surfactants.

**Table 2:** Effect of various organic solvents on proteolytic activity.

Sr. no.	Solvent	Residual activity (%)		
		Concentration of organic solvent (v/v)		
		30%	50%	70%
1	Control	100.0	100.0	100.0
2	Acetone	100 ± 0.43	98.22 ± 0.65	87.21 ± 0.13
3	Acetophenone	69.89 ± 4.35	87.84 ± 1.86	64.54 ± 1.46
4	Benzyl alcohol	80.15 ± 1.44	70.68 ± 0.27	42.44 ± 0.66
5	Benzene	95.15 ± 3.83	98.80 ± 3.57	92.19 ± 2.72
6	Butanol	63.96 ± 3.46	54.68 ± 3.56	31.68 ± 3.34
7	Chloroform	65.05 ± 2.86	57.55 ± 2.85	26.28 ± 2.35
8	Chlorobenzene	81.43 ± 2.85	100 ± 1.82	71.57 ± 2.16
9	Dichloromethane	58.52 ± 2.35	61.20 ± 2. 24	34.29 ± 2.37
10	Dimethylformamide	98.71 ± 5.97	90.13 ± 3.09	58.63 ± 3.38
11	Diethyl ether	50.16 ± 6.05	75.91 ± 4.78	61.39 ± 4.25
12	Ethylene glycol	100 ± 1.39	73.63 ± 3.06	38.14 ± 4.32
13	Ethy lacetate	58.63 ± 7.85	66.83 ± 5.85	45.51 ± 4.35
14	Methanol	71.18 ± 0.96	77.59 ± 1.75	66.83 ± 1.36
15	Propanol	84.11 ± 1.70	69.89 ± 1.58	52.11 ± 1.69
16	Tetrahydrofuran	70.37 ± 4.81	52.50 ± 7.95	18.36 ± 5.85
17	Trichloroethylene	60.81 ± 2.05	80.15 ± 2.90	70.68 ± 2.37

Data represented in average values ± SD of *n* = 3 experiment.

**Table 3:** Stability of protease enzyme preparation in various local detergents.

Sr. no.	Detergent	Residual activity (%)	
		7 mg/mL	10 mg/mL
1	Control	100	100
2	Tide	100 ± 0.20	90.38 ± 0.57
3	Ujala	100 ± 0.16	100 ± 1.38
4	Wheel	98.85 ± 0.22	98.65 ± 0.52
5	Impact	67.25 ± 0.69	65.08 ± 2.56
6	Rin	87.87 ± 0.04	85.03 ± 0.17
7	Nirma	100 ± 0.44	100 ± 0.72
8	Surf Excel	89.36 ± 0.31	83.66 ± 1.47
9	Ariel	92.49 ± 0.78	83.24 ± 0.57
10	Fena	82.54 ± 0.65	74.55 ± 0.83
11	Ghari	80.33 ± 0.11	72.48 ± 0.48
12	Sasa	78.43 ± 0.07	61.21 ± 1.26

Data represented in average values ± SD of *n* = 6 experiment.

**Table 4:** Destaining profile of different blood stains after 25 min of treatment.

Sr. no.	Treatment	Destaining efficacy (%)*				
		A	B	C	D	E
01	Control	11.89	8.69	15.91	15.66	8.74
02	Protease	0.62	0.80	0.74	0.97	0.71
03	Detergent	1.18	1.51	1.09	1.16	0.79
04	Prot. + Det.	0.24	0.12	0.26	0.10	0.24

*The intensity if blood stain at 0 min treatment was taken as 100% for all blood samples.

A: human; B: ox; C: buffalo; D: goat, and E: hen blood stained spot.

**Table 5:** Effect of protease on excision wound parameter in mice.

PWD	Wound area—percentage of wound contraction is in parentheses		
	Group I: control	Group II: soframycin	Group III: CCP
0	344.67 ± 1.75	316.22 ± 3.12	335.83 ± 1.33
4	240.53 ± 1.38 (30.22)	276.09 ± 3.01 (14.2)	222.75 ± 1.97 (33.7)*
8	162.32 ± 2.25 (52.9)	187.03 ± 1.38 (41.90)	170.89 ± 1.33 (49.7)*
12	47.67 ± 1.03 (86.17)	53.17 ± 2.04 (83.45)	38.32 ± 1.63 (88.6)*
16	27.50 ± 1.87 (92.02)	20.77 ± 1.63 (93.43)	22.67 ± 1.21 (93.29)*
20	8.33 ± 1.03 (97.58)	3.54 ± 1.87 (98.88)	4.08 ± 1.02 (98.78)*
PE	23.68 ± 1.03	19.87 ± 1.17	20.34 ± 1.37

PWD: postwounding days; values are expressed as mean **± **S.D., *n* = 6 animals in each group, PE: period of epithelialization (days), *P < 0.001 as compared to control.

**Table 6:** Antibacterial activity of crude protease.

Sr. no.	Bacterial strains	Zone of inhibition (mm)		
		CCP		Gentamicin
		50 *μ*g/mL	100 *μ*g/mL	10 *μ*g/mL
01	*Escherichia coli *	08.85 ± 0.56	13.06 ± 0.38	16.70 ± 0.60
02	*Klebsiella pneumoniae *	06.04 ± 0.49	11.24 ± 0.87	19.67 ± 0.73
03	*Pseudomonas aeruginosa *	07.67 ± 0.81	12.59 ± 0.57	18.90 ± 0.47
04	*Staphylococcus aureus *	10.34 ± 0.50	15.28 ± 2.45	20.66 ± 0.81
05	*Proteus vulgaris *	08.27 ± 0.36	14.49 ± 0.85	18.63 ± 0.47
06	*Bacillus subtilis *	06.42 ± 0.47	09.34 ± 0.45	21.16 ± 0.34

Data represented in average values ± SD of *n* = 6 experiments.

**Table 7:** Active population of root knot nematodes.

Initial population	Control	Treatment					
		Standards (2 mg/g soil sample)		CCP (mg/g of soil sample)			
		Sanjeevani	Carbofuran	2.5	5.0	7.5	10.0
2000	3826	85 (95.74)	160 (92.00)	1494 (25.34)	846 (57.65)	462 (76.91)	227 (88.65)

Values in parentheses indicate the percent mortality of nematode.

**Table 8:** Morphological observations of experimental plants on exposure of protease and standard nematicide.

Sr. no.	Plant latex	Weight (g)		Seed index	Whole plant height	Spad value	Biomass (g)*	
	(treatment)	Fruit	Seed		(cm)	(unit)	AP	UGP
1	Control	9.290 ± 2.560	0.071 ± 0.006	0.008	58.02 ± 0.356	37.85 ± 0.739	3.097 ± 0.109	0.640 ± 0.028
2	Untreated	2.079 ± 0.397	0.010 ± 0.0037	0.005	41.17 ± 1.465	28.45 ± 0.722	1.609 ± 0.056	0.425 ± 0.009
3	*T. viride* ^ ++^	4.680 ± 0.747	0.048 ± 0.010	0.010	55.20 ± 0.674	34.09 ± 1.824	3.480 ± 0.012	1.032 ± 0.020
4	Carbofuran^++^	1.850 ± 0.418	0.012 ± 0.003	0.006	51.95 ± 0.878	33.04 ± 0.415	2.729 ± 0.088	0.872 ± 0.033
5	CCP^+^	2.380 ± 0.360	0.037 ± 0.006	0.015	57.15 ± 0.788	30.12 ± 1.594	3.300 ± 0.127	0.863 ± 0.030

AP: aerial part of plant; UGP: underground part of plant; *biomass without fruit, ^+^10 mg CCP per g of soil; ^++^concentration of carbofuran and *T. viride* was 2 mg per g of soil, and data are represented in average values ± SD of *n* = 6 experiment.
